# Sedation for screening MRI in patients with congenital melanocytic naevi under the age of one is a successful, safe and economical first‐line approach

**DOI:** 10.1111/bjd.17263

**Published:** 2018-12-03

**Authors:** I. Plumptre, G. Stuart, A. Cerullo, V.A. Kinsler

**Affiliations:** ^1^ Department of Paediatric Dermatology Great Ormond Street Hospital for Children NHS Foundation Trust London U.K; ^2^ Department of Paediatric Anaesthesia and Great Ormond Street Hospital for Children NHS Foundation Trust London U.K; ^3^ Department of Paediatric Radiology Great Ormond Street Hospital for Children NHS Foundation Trust London U.K; ^4^ Genetics and Genomic Medicine University College London (UCL), Great Ormond Street Institute of Child Health London U.K

Dear Editor, A single screening magnetic resonance imaging (MRI) scan of the central nervous system with contrast in patients under age 1 year (ideally < 6 months) is currently recommended as the best predictor of adverse outcome measures in children with multiple congenital melanocytic naevi (CMN).^1^, ^2^ This recommendation is based on using sedation rather than general anaesthesia (GA), however, this practice is not routine in many departments. Recently, concerns regarding neurodevelopmental effects of GA in animals^3^ and children^4^, ^5^ have suggested that avoidance of GA is desirable where possible in infants.

We therefore undertook a retrospective analysis of the success of MRI using different modalities of sedation or anaesthesia. Records were reviewed of 247 patients with CMN who had had an MRI attempted, 161 under age 1 year. In total 114 of 247 (46%) were male, 208 of 244 (85%) had brain and whole‐spine imaging and 193 of 202 (96%) had contrast injection. The mean and median ages were 2·02 and 0·66 years, respectively (range 0–18·8 years).

**Figure 1:**
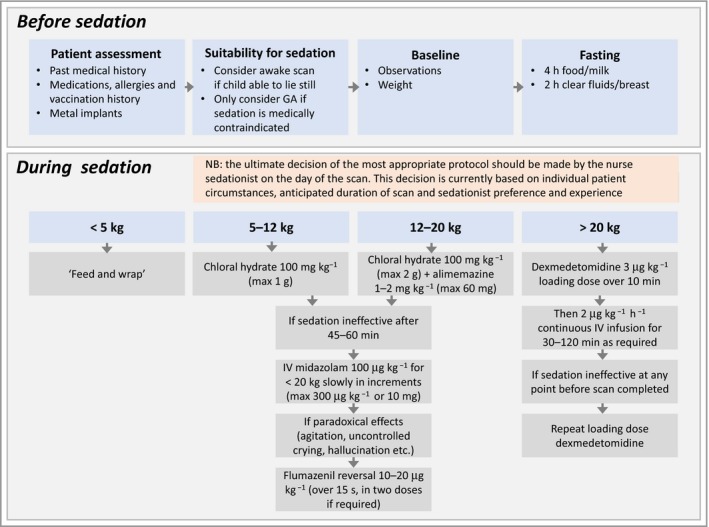
Current sedation protocol for paediatric magnetic resonance imaging used at Great Ormond Street Hospital for Children NHS Foundation Trust, London, and for which a 90% success rate was obtained in this cohort. Sedation is also cheaper than general anaesthesia (GA). IV, intravenous.

Information regarding sedation and anaesthetic was available in 208 of 247. Forty‐five of 208 (22%) were preselected for GA by an experienced sedationist, for example due to comorbidities, although a comparison of the demographic and phenotypic profiles of those who received sedation or GA showed no significant differences. Ten of 208 (5%) were awake, 151 of 208 (73%) were sedated and two of 208 (1·0%) underwent the ‘feed and wrap’ technique, all according to local protocols (Fig. 1). In total 219 of 234 scans (94%) were successfully completed, with the 15 abandoned scans all in the sedation group. This equates to a sedation success rate of 136 of 151 (90%). Regression analysis demonstrated no significant difference in sedation success by age.

The literature supports sedation for paediatric MRI as effective and safe,^6^ with comparable success rates to our results,^7^ although there is no consensus on the methods of sedation.^8^ Common minor adverse events with sedation include vomiting and excessive secretions and apnoea; serious adverse events are very rare,^6^ with none reported here. Sedation is also cheaper than GA, at £462 vs. £770 per patient.^7^

These data strongly support the use of sedation rather than GA as a first‐line approach for MRI in infants with multiple CMN, after triage by a sedationist or anaesthetist and with access to anaesthetic support if required.
